# Cognitive rehabilitation groups: A thematic analysis of feasibility and perceived benefits for clients with moderate to severe traumatic brain injury living in the Western Cape

**DOI:** 10.4102/ajod.v4i1.175

**Published:** 2015-08-20

**Authors:** Abigail Wilson, Peta Wills, Chrisma Pretorius, Leslie Swartz

**Affiliations:** 1 Department of Psychology, Stellenbosch University, South Africa; 2 Western Cape Rehabilitation Centre, Cape Town, South Africa

## Abstract

**Background:**

Traumatic brain injury (TBI) has a significant impact on the burden of care within the South African setting, impacting on the individual, the family, and the community as a whole. Often the consequences of TBI are permanent, resulting in numerous financial and emotional stressors.

**Objective:**

This research focusses on the experience of outpatient cognitive rehabilitation groups for individuals who have suffered moderate to severe brain injuries within the South African setting.

**Method:**

Participants with moderate to severe brain injury were required to attend five cognitive rehabilitation groups and engage in a semistructured interview. Qualitative data were examined via thematic analysis, to determine participants’ subjective experiences of group participation.

**Results:**

There is a need within the South African setting for cognitive rehabilitation and support groups for individuals who have experienced a TBI. The benefits were notable for both the individuals attending and their support systems. In spite of the benefits there were notable limitations to attendance, including financial restrictions and transport limitations.

**Conclusion:**

According to participants and their families, there is a scarcity of resources within the Western Cape for clients who have sustained a TBI. Despite limitations in capacity to attend there appears to be a need for structured outpatient cognitive rehabilitation programmes integrating the complex cognitive and emotional challenges faced by individuals with TBI and their families.

## Introduction

This article reports on a group intervention for survivors of traumatic brain injury (TBI) and their families. TBI is a significant public health problem worldwide (Khan, Baguley & Cameron 2003; Nell & Ormond-Brown 1991; Shukla, Devi & Agrawal 2011) and has been reported as the leading cause of death and disability in adolescents and young adults in the United States of America (Vaishnavi, Rao & Fann 2009). TBI is a prominent challenge within communities and creates a significant drain on resources for families, communities, and countries (Feigin et al. 2010), with developing countries, such as South Africa, exhibiting a higher prevalence rate in comparison to developed countries (Nell & Ormond-Brown 1991).

## Literature review

A TBI is caused by an external impact to the brain resulting in temporary or permanent neurological dysfunction (Khan et al. 2003). It is an ‘externally inflicted blow to the brain with a cause that is not of a degenerative, vascular, infectious, or congenital nature’ (Vaishnavi et al. 2009:199). According to the Department of Health in KwaZulu-Natal, South Africa, the three primary causes of TBI within the South African setting are motor vehicle, bicycle or vehicle-pedestrian accidents (accounting for over 50% of all reported TBIs), individuals falling and obtaining a TBI (accounting for an estimated 25% of reported TBIs), and finally, interpersonal violence (accounting for approximately 20% of all reported TBIs). The Department of Health in KwaZulu-Natal (2010) reported that individuals between the ages of 15 and 40 years were at the highest risk of brain injury.

TBI can cause impairment in neurological and cognitive functions leading to restrictions in activity and hindering participation in social events (Shukla et al. 2011). The consequences of TBI can be severe and permanent, altering a person’s life and causing disruption in dynamics within the family (Khan et al. 2003). Full recovery from moderate to severe TBI often does not occur, and after even two years an individual may not have reached their baseline level of functioning (Vaishnavi et al. 2009).

Functional recovery for individuals who have sustained a moderate to severe traumatic brain injury tends to be more rapid than recovery from the more complex behavioural symptoms (Vaishnavi et al. 2009). Limited capacity to participate in leisure activities and the presence of behavioural, social, and cognitive changes in individuals with TBI tends to result in increased social isolation and decreased stimulation for these clients (Laatsch, Little & Thulborn 2004; Powell & Malia 2003; Sander et al. 2012).

A brain injured individual may have reduced capacity to perform in educational and occupational settings, resulting in dependence on government issued grants for financial support. Further financial stressors are noted through increased medical expenses and loss of income generation (Radomski 2002; Sander et al. 2012; Schultz n.d.). Brain injured individuals frequently have a decreased capacity to live independently (Sander et al. 2012), and this may result in previously employed family members leaving their place of work to care for individuals, increasing financial stressors. The result is frequently a decrease, or loss, of income and earning potential, resulting in increased financial strain for the individual, and his or her family, over a lifetime (Khan et al. 2003; Sarajuuri et al. 2005). The consequences of TBI are social, emotional, and cognitive, affecting both the individual and his or her family (Gianutsos 1991).

The gaps in care for individuals (post-TBI) include both cognitive and emotional aspects, with a sample of 895 post-TBI individuals reporting that their most critical need was an improvement in cognitive skills (Heinemann et al. 2002). Cognitive rehabilitation refers to a particular set of interventions with the aim of improving an individual’s capacity to execute cognitive tasks by relearning previously learned skills and teaching compensatory strategies (Tsaousides & Gordon 2009). Cognitive rehabilitation has been defined by The American Congress of Rehabilitation Medicine as ‘a systematic, functionally orientated service of therapeutic activities that is based on assessment and understanding of the patient’s brain-behavioural deficits’ (Cicerone et al. 2000:1596). It is generally directed towards, but not limited to, attention, memory, language, comprehension, and communication and executive function (Cicerone et al. 2000; Tsaousides & Gordon 2009). Cognitive rehabilitation is the current treatment of choice for the diverse impairments that individuals with TBI experience and is currently standard practice worldwide (Sander et al. 2012).

Current literature indicates that cognitive rehabilitation programmes have been shown to be effective in restoring some physical and vocational functioning (Kim 2011). Improvement in individual and family financial status and emotional well-being has been reported following participation in cognitive rehabilitation programmes (Sander et al. 2012). Cognitive rehabilitation groups generally aim towards increasing a person’s capacity to integrate effectively with his or her community which includes, amongst other things, being accepted by others, being a part of a network of family and friends, and being involved in meaningful and productive day to day tasks (Winkler, Unsworth & Sloan 2006). With participation in cognitive rehabilitation groups and the provision of homework tasks, to be completed with family members, one aims to increase participants’ sense of belonging and sense of purpose within society.

Very little is known about cognitive rehabilitation groups and their use in Africa (Nell & Ormond-Brown 1991; Schneider et al. 1999; Schrief et al. 2008). The current study fills a gap in the literature by providing a description of this type of intervention in a low resource context.

## Objectives

The aim of this study was to describe a pilot outpatient cognitive rehabilitation programme for individuals with a moderate to severe traumatic brain injury. The research aims to determine whether cognitive skills programmes were perceived as subjectively beneficial, both cognitively and emotionally, by participants.

## Methodology

The research utilised a qualitative research paradigm to extract predominant themes relating to the subjective experiences of group participants and their caregivers. A qualitative research methodology allows space for exploration into the quality and texture of experience and therefore embraces the individuality of the participants’ experiences (Hesse-Biber & Leavy 2006). The interviews were qualitatively understood through utilising thematic analysis techniques, which is understood as ‘a method for identifying, analysing and reporting patterns or themes within data’ (Braun & Clarke 2006:79). This is achieved by organising the data into thematic groups in order to understand and describe subjective experience. Data were gathered within the group setting (recorded by note taking immediately after each session) and in individual semistructured interviews following participation in five group sessions. A.W. and P.W. were involved in both the intervention and the data collection. P.W. is a qualified clinical psychologist working in the field of rehabilitation and A.W. was a master’s student in psychology with considerable experience in the counselling field.

The research recruited individuals with moderate to severe TBI to participate in the groups and individual interview process. They were recruited from a specialised rehabilitation centre within the Western Cape via purposive sampling. It was required that suitable candidates needed to have sustained a moderate to severe brain trauma based on a Glasgow Coma Scale (GCS) score of less than 13 on admission to hospital and/or post-trauma amnesia lasting for a period longer than 24 hours postinjury (Edeling et al. 2013). Research indicates that the plasticity of the brain becomes notably limited 24 months postinjury and for this reason time of injury was restricted to within the past 24 months (Ribbers 2013). All candidates were required to speak conversational English and at least one responsible adult needed to be available in order to assist them in attending the group and with postgroup tasks. Individuals with psychiatric illness, previous brain injury, or significant language impairments were excluded from the sample due to participation limitations and confounding variables.

Written consent using an approved form to attend the groups and participate in the individual interview process was received from all participants (*N* = 14) as well as from their primary caregivers (who accompanied them to sessions), where applicable. This represented all those who attended the groups. Those who provided consent were required to participate in five once weekly groups which were divided into domains of cognition. These domains were based on previous divisions created by Powell and Malia (2003), and included education regarding brain injury, memory skills, thinking skills, executive skills, and awareness skills. In addition, participants were required to complete homework tasks outside of the group, encouraging learning and application of skills covered within the session and increased interaction with family members. Finally, consent to participation required that clients engage in an individual semistructured interview on completion of the group intervention to ascertain their experiences within the group process. Participants’ age range was from 19–67 years with a mean age of 31.7 years and standard deviation of 16.7 years, representing a cross section of individuals identified as ‘at risk’ for TBI within the South African setting (Nell & Ormond-Brown 1991). Time of injury ranged from between six and 24 months, and brain injury location and cause of injury was variable across the sample. Participants varied in terms of sex, ethnicity, primary language and level of education (Table 1).

**TABLE 1:** Participants’ demographic characteristics.

Participant code	Age	Gender	Race	Home language	Level of education
T.W.	66	Female	Indian	English	Grade 12
G.P.	19	Male	Mixed-race	Bilingual	Grade 10
J.C.	19	Male	Black	isiXhosa	Grade 12
N.F.	20	Female	Mixed-race	Bilingual	Grade 11
P.D.	67	Female	Mixed-race	Bilingual	Grade 9
L.D.	22	Female	Mixed-race	Afrikaans	Grade 12
H.M.	24	Male	Mixed-race	English	Grade 12
R.L.	18	Male	Black	isiXhosa	Grade 9
B.A.	51	Male	Mixed-race	Bilingual	Grade 12
K.C.	24	Male	Black	isiXhosa	Grade 12
D.D.	31	Male	Mixed-race	English	Grade 12
Y.M.	19	Male	White	Afrikaans	Grade 11
R.C.	54	Male	White	English	Grade 12
G.M.	38	Male	Mixed-race	Bilingual	Grade 8

### 

#### Ethical considerations

Ethical approval for the study was obtained from the Human Subjects Ethics Committee at Stellenbosch University, from the Department of Health in the Province, and from the Hospital itself. All participants were fully informed of their rights, including their right to withdraw at any time. No refusals were obtained. The participants’ confidentiality was protected by assigning them a participant code when the data was analysed.

## Findings and discussion

Analysis of the data revealed that despite some differences, there were numerous shared attitudes, thoughts and opinions identified across all interviews. Aspects of identity and separation from society dominated the groups, with the group serving as a transition vessel for acceptance by others.

### The preinjury and post injury self

A predominant theme within the analysis was the shift, or loss, of identity following brain injury. With a perceived ‘fault line’ between the person before and the person after the brain injury, resulting in a sense of alienation from the person after injury. Within this came a sense of eroded memories and a desire to preserve the previous self, as expressed by Y.M.: ‘They all remember me the way I was. I didn’t want them to see me the way I am now’. This need for preservation of identity prior to injury seemed necessary for the brain injury survivor but also presented as a strategy to assist others in coping with the changes and inherent loss of some dreams and expectations held by family members. In discussing families coping with changes postinjury this adjustment of expectations became salient. R.C. shared this sentiment during one of the sessions together: ‘when I had the accident she [*mother*] cried because I’m not the boy I was meant to be’. Paterson and Scott-Findlay (2002) further link this theme with the challenges of limited self-awareness and insight which many individuals experience after traumatic brain injury, which further contaminates the individual’s journey towards reintegration of the self.

### A sense of exclusion

The theme of feeling existentially lost due to changes in the self, as well as forgotten by society, permeated the group and individual sessions. This is consistent with past research of brain injury survivors which indicates that social isolation and reduced participation in social activities is a common consequence of brain injury (Sander et al. 2012; Winkler et al. 2006). G.M. shared this feeling of isolation during the sessions: ‘I was lonely at the time when I had the accident … I was always just basically on my own. … I felt so helpless’. A sense of loss of connection to the world seems to be a common experience amongst brain injury survivors and a desperate need to go back to how it was, to reconnect, and feel a sense of belonging again. This sense of disconnection and change was expressed frequently within the group sessions and shared again by P.D. in the individual interview: ‘It’s been difficult for me. When I had the stroke, when I had the stroke – then I had many friends, but my friends didn’t come and visit me’. Difficulties with sustained attention and fatigue further exacerbate the challenges of societal reintegration through decreased tolerance and an increased sense of confusion in high stimuli environments (Paterson & Scott-Findlay 2002). A sense of wanting to change, either back to who one was or to rediscover the self, and adapt to changes post-trauma in order to again be a part of society, was apparent. B.A. expressed this perception of change and displacement during the sessions: ‘You are just you and out there is people that want you to be like them’. Yet within this theme participants expressed a lack of skills and understanding to engage in this process of change and reconnection, which presented as a significant hindrance to their recovery process. Social exclusion post-traumatic brain injury, and a lack of skills to include the self or relearn social skills in isolation, seemed to result in a further loss of social skills, and hence increased social exclusion. It seemed that many individuals with traumatic brain injury eventually isolate themselves entirely from society due to an increasing sense of social incompetence and exclusion as well as decreasing self-esteem. As G.M. stated, more than one year postinjury: ‘Nowadays, I’m just like … like … being at home, staying at home, and I don’t usually go out with my family’. The sense of loneliness and isolation from society, experienced by participants, permeated many of the group sessions. Thus, it is not surprising to find such high rates of both depression and anxiety amongst people who have experienced traumatic brain injuries (Tsai et al. 2014). The group seemed to serve as a valuable place to allow for participants to reintegrate into society through allowing them to interact socially and assist other group members when required. Y.M. shared this sense of reinclusion during a session together:

‘I used to, because of the accident, I used to shy away from conversation with people … I can speak with people more now by getting to interact … by saying more.’

(Y.M.; 19-year-old male)

### Reintegration of the self through others

The concept of cognitive rehabilitation tends to focus on skills of memory, language, executive processes, and other aspects related specifically to how one thinks and reasons in day to day living. The rehabilitation of cognitive functions post-traumatic brain injury rarely acknowledges the importance of the client’s need to, in a sense, rehabilitate their own identity; to redevelop and relearn their own identity and the implications of this on themselves, their families, their communities, and their futures. The groups offered in this intervention did not provide a specific space for this existential exploration, yet within the tasks of memory, thinking, executive, and language skills, the group seemed to allow for the rediscovery of the self. As Y.M. shared during the sessions, in regards to re-establishing contact with friends and family which he had isolated himself from since the accident: ‘I think I must communicate with them, because I’m not shy for people to see how I am now anymore’. The sense of increased confidence through the development of personal identity post-traumatic brain injury was apparent within the group interventions.

Although the more traditional cognitive tasks were beneficial to the participants, the predominant theme that emerged was that the groups allowed for interaction and exploration within the safe and reflective setting in order to begin rediscovering the self. For many participants the group setting was the first social interaction they had experienced that was non-judgemental and supportive, which allowed them to explore their strengths and weaknesses postinjury within a space where they felt they belonged. Being surrounded by people who had experienced brain trauma allowed clients to interact more freely. B.A. expressed this sentiment during the individual interviews after the group sessions had run their course: ‘Because every human being is different like. But, but we are the same. We are a family. We have become a family’. The group developed into a space where the participants could access their identity prior to their injuries and begin the process of integrating that with their present identity, providing a space to belong and explore the self. Within this the groups seemed to break the cycle of social exclusion and declining self-esteem. In reflecting on the group experience R.C. was able to verbalise this sense of personal development: ‘It changed my life and made me a better person and it made me that I don’t feel so bad about myself anymore. Not like I did before’.

### Birth of identity

The cognitive rehabilitation groups seemed to provide a space for participants to regain their sense of self through a journey of learning and self-discovery. The groups allowed for increased insight in terms of cognitive strengths and weaknesses as well as interpersonal and emotional development. Y.M. described this experience during individual interviews:

‘It was talking about what happened to me … sharing with the other people that were in the group with me. And because I see there are other people that went through or similar incidents that I went through and they are going on with their lives. It has actually encouraged me also to not sit back and to go on with my life.’

(Y.M.; 19-year-old male)

The group participants encouraged one another to explore themselves and rediscover who they are postbrain injury. When leaving the group, B.A. expressed how this changed his perceptions of himself as an individual, as a unique being, separate from the perceptions of society:

‘Every time when I walked out here, I said to myself, “from now on what you learn today, you must be yourself. Don’t let other people around you take over your life. Don’t let them climb into you. Remember you be you.”’

(B.A.; 51-year-old male)

The desire for participants to connect with others and interact was overwhelming with the majority of members requesting ongoing groups and an increased frequency of the groups.

### Building bridges and creating confidence

The group served as a bridge to various societal links, to one another within the group and creating new links between caregivers and survivors. As groups progressed, involvement by family members seemed to increase and there was a sense of pride expressed by the caregivers at the capacity and enthusiasm that their family member exhibited whilst engaging in the cognitive tasks provided to them. In response to a question probing this shift in family and caregiver relationships, B.A. was able to share:

‘I think the reason why is because I’m doing stuff now on my own and my wife and daughter say, “Aah, look what he has done, hey? You can see him going better and better, better now!” Then I feel so proud of myself that I burst out in tears.’

(B.A.; 51-year-old male)

The groups provided a starting-point for the development of skills and confidence which participants were able to generalise across setting, allowing for improved interpersonal relationships, increased self-confidence, and an overall improvement in perceived quality of life.

Research by Sander et al. (2012) found a direct relationship between the emotional functioning of caregivers to TBI survivor’s productivity and social integration. The current research indicates that this relationship is interdependent and reciprocal, such that as one factor improved others also strengthened and improved allowing for notable changes within the entire family systems. A change was perceived during the group interventions, as families began feeling a sense of pride at the accomplishments and determination of their spouse or child; as B.A. shared, ‘they [*family*] are very, very, proud as well … I am told that I am getting on well now. Better than before …’, and participants seemed to flourish at the opportunity to have goals, an identity, and a sense of responsibility. Similar findings were noted in the research by Bay, Blow and Xie (2012), showing that increased involvement with others and a sense of being valued within a group had a positive impact on emotion regulation, confidence, and social skills.

The increased support by caregivers, family and friends, through participation in a group, seemed to allow for increased confidence amongst group members and an increased desire to accomplish more in order to further enhance relationships. Y.M. shared this newfound sense of confidence and energy: ‘I actually go out more now. Before I didn’t want to go out’. In this sense, the group sessions allowed participants to begin exploring beyond the group setting, beyond the safety of the family setting, and into the community, which had previously felt to be a scary and rejecting place. G.M. was able to express this change in societal perceptions following participation in the rehabilitation groups: ‘my friends tell me I’m a little better … They are respecting me now’.

The act of participating in a cognitive rehabilitation group seemed to have an impact far beyond improving the initially identified cognitive skills of memory, executive functioning, language and attention. Although these aspects featured as important in providing clients with the skills to interact socially, they were overshadowed by the intrapersonal and interpersonal benefits of group participation: ‘Now, I speak more to my Mom. Before I never used to speak a lot to her’ (Y.M.). The groups allowed for the cycle of social exclusion and decreased self-esteem to be broken and allowed participants to develop their own identity again postinjury. Statements made by the participants such as, ‘I believe in myself now’; ‘My mood is more up than down now. Before I used to be very depressed’ (Y.M.); and ‘I’m now more calm and at ease’ (G.M.), are indications of the value of these interventions in assisting individuals who have experienced traumatic brain injuries.

### Practical skills developed

The group aimed to explore the experience of cognitive rehabilitation groups within a low socioeconomic setting. Although the predominant themes of identity and a desire to belong within society emerged from the data analysis, there were also clear cognitive benefits of the group expressed by the participants. Concrete skills and compensation strategies served as useful tools to assist clients in developing their social abilities and interpersonal skills. Y.M. was able to identify his limitation and implement compensatory strategies based on the skills learnt:

‘I got a diary to help me remember things because my short term memory is not so good. Sometimes I forget so I write in the diary. So if I do forget then I look at it and I read what I did that day and then I remember. Now I can look back and tell them.’

(Y.M.; 19-year-old male)

Cognitive skills allowed for participants to engage more appropriately with individuals and to better recall what they needed to do as well as what friends and family members were doing. Participants reported an improvement in both memory skills and executive skills following the brief cognitive rehabilitation group intervention, as B.A. shared, ‘The main thing what I have learned here is to make that plan and how to do that plan’. In addition, the group provided clients with a new sense of motivation and confidence to practice and learn new skills in the future in order to further improve their quality of life: ‘Now I actually practice my handwriting more since I have been in the group. And I read more. So it has encouraged me to read more’ (Y.M.). The groups also provided concrete techniques to assist participants, and family members were encouraged to engage with the members in order to better understand brain injury. Through improving understanding, family members were better able to assist in the growth of participants, which further increased confidence; as Y.M. shared, ‘She used to laugh before when I said that I couldn’t remember. Now she helps me to remember things’. The homework aspect of the groups enhanced the development of connections amongst family members and caregivers, further contributing to the theme ‘building bridges’.

Participants expressed notable cognitive benefits from participating in the groups but the group sessions also seemed to provide a space beyond ‘cognition’, serving as a base to reconnect with the self and society and thus improving the interpersonal relationships for group members.

## Conclusions

The subjective experiences of participants within this group process was overwhelmingly positive, with an increased sense of belonging within society, and increased understanding of the self, as well as an improvement in some cognitive skills, such as memory and planning. In spite of logistical challenges, participants managed to attend the majority of sessions and the reality of the challenges they faced became integral to the group process.

Suggestions by participants were to include more people in the groups and to include more homework tasks that required access to the community: the presence of multiple languages and individual differences in functional levels presented as a constant challenge within the groups, as expressed by G.M. in the interviews: ‘Some words I could never understand, but … I didn’t had [*sic*] a dictionary which I could have looked it up in the dictionary’. The multiple languages used within South Africa are something one needs to take cognisance of when preparing group interventions.

The end of the group marked the end of a valuable journey for all participants and their discontinuation presented as a significant loss to many members: ‘It feels like it is the end of beautiful things’ (B.A.). Further research is required within the South African and Africa setting to determine the applicability of cognitive rehabilitation groups amongst this population (Nell & Ormond-Brown 1991; Schneider et al. 1999; Schrief et al. 2008). There is a notable need within the South African and African setting to improve post-TBI interventions in order to enhance recovery outcomes (Feigin et al. 2010; Marquez de la Plata et al. 2008; Schalock 1998). This study provides tentative evidence that it is possible to mount such a programme with scant resources; more information from other contexts will help build a stronger evidence basis.
